# ST-FGDIR: Spatiotemporal Traffic Forecasting with Forecast-Gain-Guided Deviation–Invariance Routing

**DOI:** 10.3390/s26196319

**Published:** 2026-10-07

**Authors:** Qianxin Xie, Yancun Jiang, Jinfeng Xu, Yuxuan Zhang

**Affiliations:** 1 School of Information Engineering, Guilin University, Guilin 541006, China; xieqianxin@glc.edu.cn (Q.X.); yancunjiang@glc.edu.cn (Y.J.); 2 College of Intelligent Science and Engineering, Beijing University of Agriculture, Beijing 102206, China; 3 Department of Computer and Electrical Engineering, Mid Sweden University, 85170 Sundsvall, Sweden

**Keywords:** traffic forecasting, spatiotemporal graph neural networks, deviation-aware learning, forecast-gain-guided routing

## Abstract

Accurate multi-step traffic forecasting requires models to respond to genuine traffic-state transitions while resisting deviations caused by missing or noisy observations. Existing deviation-aware methods characterize departures from recurrent patterns, but deviation magnitude alone does not indicate whether the resulting correction will improve a node-wise forecast. Guided by the insight that correction strength should depend on forecast gain rather than deviation magnitude, we propose Spatiotemporal Traffic Forecasting with Forecast-Gain-Guided Deviation–Invariance Routing (ST-FGDIR). ST-FGDIR constructs an anchor-conditioned stable endpoint and a deviation-corrected candidate through weight-shared current–anchor graph encoding and multi-view deviation evidence. Signed candidate-error differences provide soft forecast-gain targets for a node-wise continuous router, while selective risk extrapolation and a false-activation loss control stable-endpoint risk across perturbation environments and suppress perturbation-induced increases in residual injection. Experiments on METR-LA, PEMS-BAY, PEMS04, and PEMS08 show competitive forecasting performance relative to published baseline results. The inference gates exhibited Spearman correlations of 0.49–0.53 with realized correction gain, and the complete model yielded an average relative MAE increase of 9.38% across 12 controlled dataset–perturbation conditions. These results demonstrate that forecast-gain-guided routing coordinates stable periodic prediction with adaptive deviation correction under both clean and perturbed inputs.

## 1. Introduction

Accurate multi-step traffic forecasting supports signal control, congestion management, and network-wide mobility planning. Traffic observations form graph-structured time series in which conditions propagate across connected roads while daily and weekly rhythms generate recurring temporal patterns. Graph recurrence, adaptive dependency learning, memory, attention, self-supervision, and dynamic graph modeling have progressively strengthened spatiotemporal representation learning [1,2,3,4,5]. Real traffic, however, continually alternates between regular patterns and transient departures caused by genuine state transitions, missing observations, sensor noise, or weak historical support.

We study the node-wise marginal utility of a deviation-derived residual relative to an anchor-conditioned stable endpoint. The resulting stability–adaptivity problem has two limiting cases: suppressing the residual can miss informative transitions, whereas applying it indiscriminately can propagate unreliable departures into future predictions.

Recent methods characterize nonstationarity through normalization or trend, seasonal, and frequency decomposition [6,7,8], self-supervised deviation and heterogeneity learning [4,9], and explicit separation of periodic and aperiodic events [10]. Online adaptation and invariant learning further address evolving distributions and environment-dependent variation [11,12,13,14]. These approaches improve deviation representation or predictor stability, but the unresolved quantity is the sample–node-level gain produced by applying a particular residual correction.

**Figure 1:**
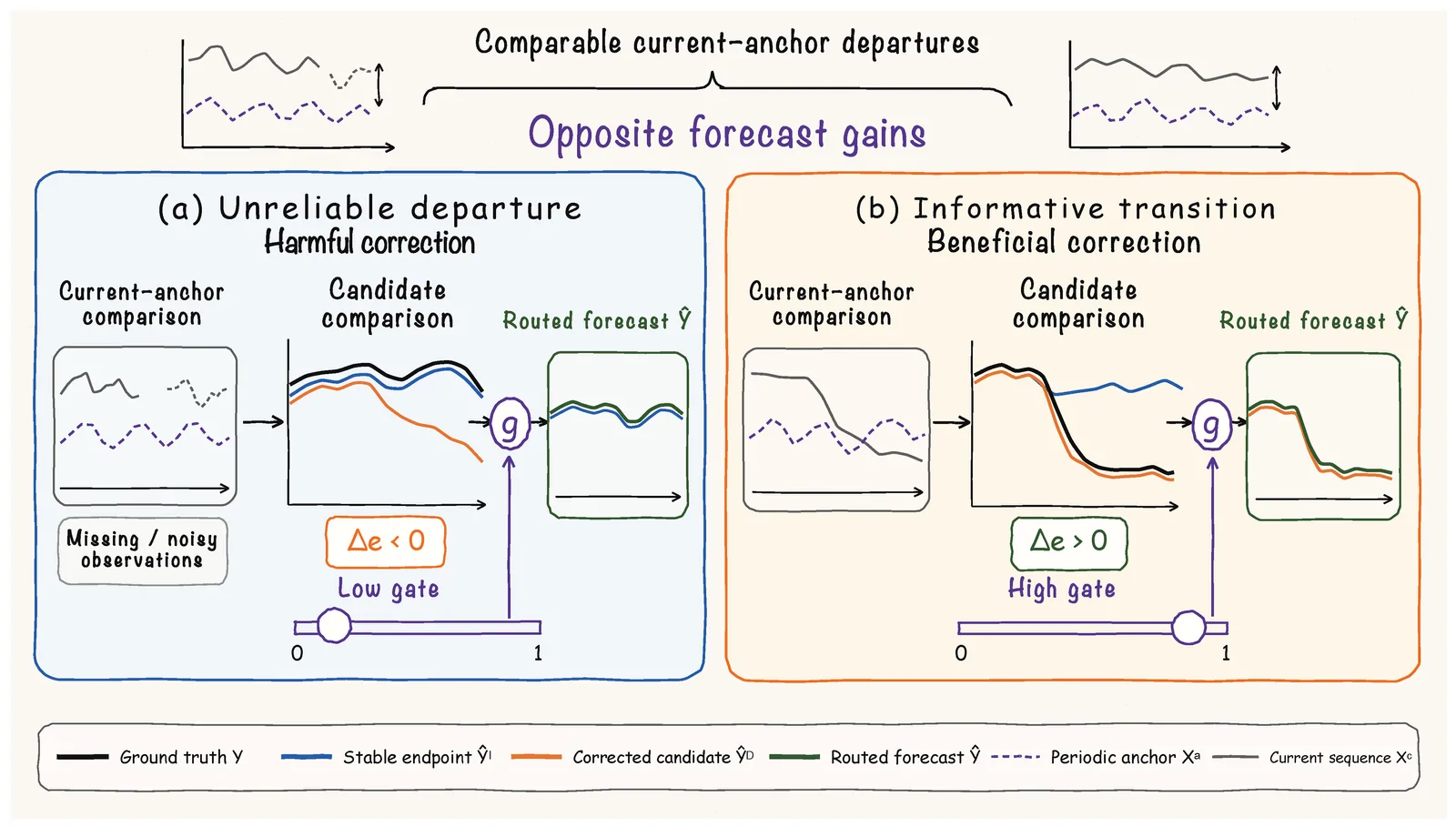
Motivation for forecast-gain-guided deviation routing. Comparable current–anchor departures can have opposite correction utilities. (**a**) When the deviation-corrected candidate increases forecasting error (Δe<0), a low gate preserves the stable endpoint. (**b**) When the correction reduces forecasting error (Δe>0), a high gate assigns greater weight to the deviation-corrected candidate. The continuous gate g∈(0,1) interpolates between the two candidates.

We address this gap by deriving correction-utility supervision from paired forecast candidates and using the resulting signal for residual routing and selective cross-environment stabilization. Figure 1 summarizes the key insight. Comparable current–anchor departures may have opposite utilities; harmful corrections should receive low gates that preserve the stable endpoint, whereas beneficial corrections should receive larger residual weights.

We therefore propose Spatiotemporal Traffic Forecasting with Forecast-Gain-Guided Deviation–Invariance Routing (ST-FGDIR). A weight-shared GraphGRU encodes the current sequence and a training-fitted weekly anchor, producing an anchor-conditioned stable endpoint. Hidden-state, statistical, spatial, prototype-distribution, and reliability evidence drives a deviation residual proposal and its corrected candidate. During training, the signed error difference between the paired candidates forms a soft forecast-gain target. A node-wise, horizon-shared router predicts this target from inference-available evidence and continuously controls residual injection. Selective risk extrapolation regulates the mean and cross-environment variation of stable-endpoint risk, where the clean target favors the stable path, while false-activation regularization discourages perturbations from spuriously increasing residual weights. A staged schedule coordinates stable forecasting, deviation modeling, router learning, and joint robustness optimization.

Experiments on METR-LA, PEMS-BAY, PEMS04, and PEMS08 show competitive forecasting performance relative to published baselines, positive gate–gain rank associations across datasets, and smaller relative MAE increases than ST-SSDL and the ablated variants across all 12 controlled dataset–perturbation conditions.

The main contributions are summarized as follows:

- We construct an anchor-conditioned stable endpoint and a deviation-corrected candidate, using their signed error difference to form a soft supervision target for continuous, node-wise residual routing.
- We couple residual routing with selective stabilization by using the clean forecast-gain target to weight stable-endpoint risk across perturbation environments, together with false-activation regularization.
- We evaluate forecasting accuracy, gate–gain alignment, and perturbation stability on four traffic benchmarks, with ablations of residual routing and robustness constraints.

## 2. Related Work

### 2.1. Spatiotemporal Representation Learning for Traffic Forecasting

Traffic observations form graph-structured sequences in which road connectivity and latent interactions jointly govern state propagation. DCRNN embeds diffusion graph convolution within a recurrent architecture to model directed spatial propagation and temporal evolution [1], whereas STGCN couples graph convolution with temporal convolution to extract localized spatiotemporal patterns [15]. Graph WaveNet combines adaptive adjacency learning with dilated causal convolution to capture latent spatial relations and long-range temporal dependencies [2]. MTGNN further integrates graph learning, mix-hop propagation, and multiscale temporal convolution [16], while AGCRN generates node-adaptive graph-convolutional and recurrent parameters from node embeddings [17]. Together, these models established the transition from propagation over a prescribed road graph to data-adaptive spatial dependency learning.

Subsequent studies enriched traffic representations with node identity, dynamic structure, long-range propagation, memory, and spatial heterogeneity. STID demonstrated the predictive value of spatial and temporal identity embeddings [18], and MegaCRN retrieved representative traffic patterns through a memory-augmented meta-graph [3]. D2STGNN decoupled traffic signals into diffusion and inherent components [19], whereas PDFormer modeled propagation delay together with dynamic long-range dependencies [20]. TESTAM combined time-enhanced attention with expert routing supervised by regression-error-based pseudo-labels to select among predictors with different spatial modeling mechanisms [21]. Memory augmentation and heterogeneity-aware graph recurrent modeling have also been used to encode spatiotemporal context and differentiated dependencies among traffic nodes [22,23].

Collectively, these methods strengthen spatiotemporal representations or adaptively select among complete predictors.

### 2.2. Deviation-Aware and Robust Traffic Forecasting

Traffic sequences exhibit nonstationarity through periodic drift, trend changes, and localized transitions. ST-Norm normalizes spatial and temporal components separately to reduce their statistical discrepancy [6], while ST-WA adapts spatiotemporal parameters to evolving traffic patterns [24]. Explicit decomposition has been pursued by STDN, which models interacting trend and seasonal components [7], and by RIFT-STGNN, which interleaves frequency- and trend-domain spatiotemporal modeling [8]. Periodic shift and event-aware graph convolution incorporates calendar shifts and external events into congestion prediction [25]. ST-SSDL instead learns the deviation between current states and historical patterns through self-supervised objectives and uses this information for spatiotemporal forecasting [9].

Self-supervised and masked pre-training offer a complementary route to learning representations from diverse traffic contexts. ST-SSL combines spatial and temporal self-supervision to capture local and global dependencies for traffic-flow prediction [26]. STD-MAE decouples spatial and temporal masking objectives so that transferable structure can be learned from partially masked sequences [27]. These objectives improve the encoded representation, while the downstream forecast remains governed by a common decoding mechanism.

Distribution-shift research addresses both stable representation learning and out-of-distribution risk identification. At the graph-representation level, GIL learns invariant graph features, while EERM optimizes graph environments to identify stable predictive relations [28,29]. Complementary work in multi-sensor monitoring attaches posterior uncertainty scoring to frozen predictors for identifying out-of-distribution inputs [30]. Risk extrapolation provides a related optimization principle by jointly controlling average risk and its variation across observed environments [11]. Together, these approaches connect robustness to stable predictive risk and explicit awareness of inputs beyond the training distribution.

For spatiotemporal forecasting, DIDA models dynamic graphs under spatiotemporal distribution shifts [31], and distribution-shift-aware urban prediction explicitly accounts for spatial and temporal environmental variation [32]. Related multi-sensor monitoring work uses dynamic causal graphs to suppress spurious inter-sensor dependencies and improve robustness under noise and changing operating regimes [33]. CauSTG captures invariant relations across spatiotemporal environments [12], whereas CaST uses a causal formulation to separate stable and environment-dependent factors [34]. STONE addresses simultaneous spatial and temporal out-of-distribution shifts [13], and DIST improves robust traffic forecasting through disentangled spatiotemporal representations [35].

ST-SSDL learns representations of current–historical deviations [9], whereas ST-FGDIR supervises the utility of applying a specific residual correction. Unlike TESTAM’s selection among expert predictors [21], our router continuously scales a residual relative to an anchor-conditioned stable endpoint, using a soft target derived from the paired candidate-error difference. The same target, computed on clean inputs, also weights stable-endpoint risk across perturbation environments. This gain-based weighting makes the REx objective [11] selective with respect to correction utility and connects stable-path regularization with residual routing.

## 3. Problem Definition

Let $G=(V,E,\tilde{A})$ denote a traffic sensor graph, where V and E are the node and edge sets, N=|V| is the number of sensor nodes, and $\tilde{A}\in R^{N\times N}$ is the normalized static adjacency matrix. For a mini-batch of size *B*, the traffic history and its observation mask are denoted by $X^{c}\in R^{B\times T\times N\times 1}$ and $M^{c}\in {\left\{0,1\right\}}^{B\times T\times N\times 1}$, respectively, where *T* is the historical input length. Under the benchmark null-value convention, $M_{btn}^{c}=1$ marks a finite, nonzero traffic measurement for sample *b*, time step *t*, and node *n*. The prediction target is a univariate traffic variable, and the feature dimension of each input and output tensor is one.

Each input window is paired with a time-aligned periodic anchor $X^{a}\in R^{B\times T\times N\times 1}$ and anchor statistics $S^{a}=\left\{S^{\mathrm{mad}},S^{\mathrm{std}},S^{\sup}\right\}$, which contain the median absolute deviation (MAD), standard deviation (STD), and historical support. The model also receives historical temporal covariates $T^{x}$, known future time-of-day covariates $T^{y,\mathrm{tod}}$, and the normalized graph, while learnable node embeddings form part of the model parameters. The forecasting map is

(1) $$\hat{Y}=f_{\Theta }\;\left(X^{c},M^{c},X^{a},S^{a},T^{x},T^{y,\mathrm{tod}},\tilde{A}\right)\in R^{B\times H\times N\times 1},$$

where *H* is the forecast horizon, Θ collects the trainable parameters, and $Y\in R^{B\times H\times N\times 1}$ is the future traffic sequence used for training and evaluation.

The periodic anchor table and its statistics are estimated once from the complete training period and then fixed for all training, validation, and test windows.

ST-FGDIR jointly models the deviation of the current state from its periodic anchor and the forecast gain of the corresponding correction. An anchor-conditioned stable path produces the stable endpoint ${\hat{Y}}^{I}$, which denotes the complete *H*-step candidate forecast, and a deviation path produces a residual correction ${\hat{R}}^{D}$. The superscripts *I* and *D* denote the selectively stabilized and deviation-corrected paths, respectively. Their sum forms the deviation-corrected candidate

(2) $${\hat{Y}}^{D}={\hat{Y}}^{I}+{\hat{R}}^{D}.$$

For each sample–node pair, the model learns a continuous routing gate $g_{bn}\in \left(0,1\right)$ shared across forecast horizons. The routed forecast is

(3) $$\hat{Y}={\hat{Y}}^{I}+g⊙{\hat{R}}^{D},\;g\in {\left(0,1\right)}^{B\times 1\times N\times 1}.$$

Values of $g_{bn}$ near zero retain the stable endpoint, whereas values near one apply the complete residual correction. The node-wise gate scales the deviation residual according to its estimated forecast gain.

## 4. Methodology

### 4.1. Framework Overview

**Figure 2:**
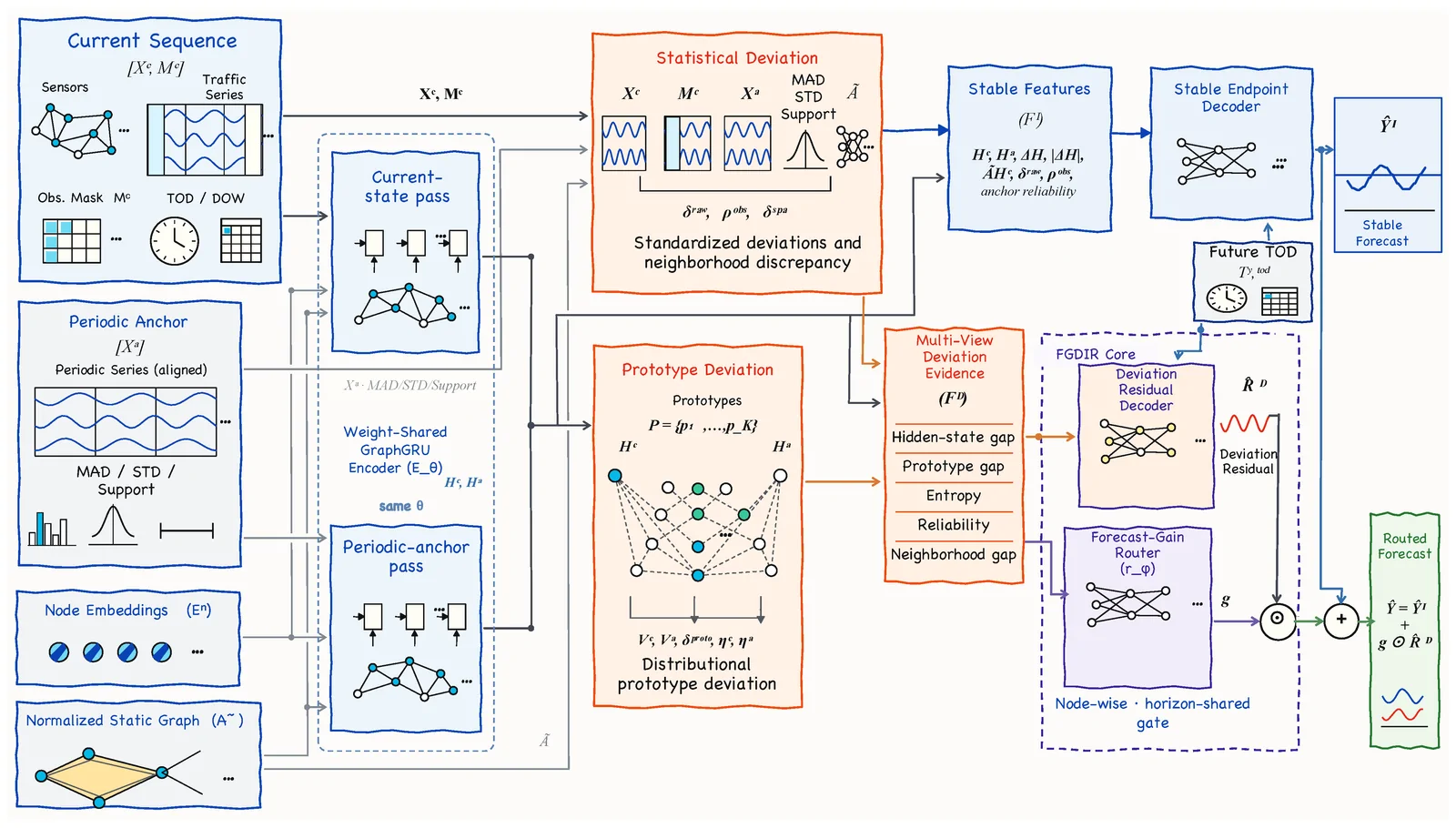
Overall architecture of ST-FGDIR. A weight-shared GraphGRU encodes the current sequence and its periodic anchor. Anchor-conditioned features generate a stable endpoint, while multi-view deviation evidence serves as input to the deviation residual decoder and Forecast-Gain Router. Ellipses denote repeated sequence elements, nodes, or network units; ⊙ and + denote element-wise multiplication and addition, respectively.

Figure 2 presents the ST-FGDIR architecture. A weight-shared graph-convolutional gated recurrent unit (GraphGRU) [1] encodes the current traffic sequence and its periodic anchor, producing node states $H^{c}$ and $H^{a}$ in a common representation space. The model then extracts current–anchor deviation information from traffic values, hidden states, prototype distributions, observation reliability, and spatial neighborhoods.

The anchor-conditioned stable path generates ${\hat{Y}}^{I}$ from paired hidden states and statistical reliability features and is regularized across perturbed environments by the selective risk extrapolation (sREx) objective. The deviation path decodes multi-view evidence into ${\hat{R}}^{D}$. The Forecast-Gain Router estimates the node-wise forecast gain of this correction and uses a continuous gate to produce Equation (3).

### 4.2. Training-Derived Periodic Anchors and Shared Graph Encoding

Let d(t)∈{0,…,6} denote the day of the week and s(t)∈{0,…,P−1} the within-day slot of timestamp *t*, where *P* is the number of slots per day. Timestamp *t* is assigned to the weekly calendar cell

(4) κ(t)=Pd(t)+s(t).

For calendar cell *k* and node *n*, the valid observations and their support over the complete training period are

(5) $$C_{kn}^{\mathrm{tr}}=\left\{\tau :0\leq \tau <T_{\mathrm{tr}},\;\kappa \left(\tau \right)=k,\;O_{\tau n}=1\right\},\;S_{kn}^{\sup}=\left|C_{kn}^{\mathrm{tr}}\right|,$$

where $T_{\mathrm{tr}}$ is the exclusive end index of the training period and $O_{\tau n}=1$ marks a finite, nonzero observation. For $S_{kn}^{\sup}>0$, the cell mean, median, MAD, and STD are

(6) $$\begin{array}{cccccc} \mu_{kn} & = & \frac{1}{S_{kn}^{\sup}}\sum_{\tau \in C_{kn}^{\mathrm{tr}}}X_{\tau n}, & m_{kn} & = & {\mathrm{median}}_{\tau \in C_{kn}^{\mathrm{tr}}}X_{\tau n},\\ S_{kn}^{\mathrm{mad}} & = & {\mathrm{median}}_{\tau \in C_{kn}^{\mathrm{tr}}}\left|X_{\tau n}-m_{kn}\right|, & S_{kn}^{\mathrm{std}} & = & {\left[\frac{1}{S_{kn}^{\sup}}\sum_{\tau \in C_{kn}^{\mathrm{tr}}}{\left(X_{\tau n}-\mu_{kn}\right)}^{2}\right]}^{1/2}. \end{array}$$

For position *t* in sample *b*, the aligned anchor is $X_{btn}^{a}=\mu_{\kappa (t_{bt}),n}$, where $t_{bt}$ is the corresponding absolute timestamp. A zero-support cell–node pair uses the node-wise training median as its anchor and the node-level training MAD and STD. A node with no valid training observation uses the global training median, with its MAD and STD set to zero. The support remains zero in both cases.

Invalid current observations are filled with training-derived node medians, with the global training median used for nodes without valid training observations. The filled current sequence and the anchor sequence are normalized using training-set statistics, while the original observation mask is retained. At each historical step, the encoder receives the normalized traffic input, its observation indicator, cyclic temporal encodings, and a learnable node embedding:

(7) $$u_{btn}^{c}=\left[X_{btn}^{c}∥M_{btn}^{c}∥\sin\left(2\pi T_{bt}^{\mathrm{tod}}\right)∥\cos\left(2\pi T_{bt}^{\mathrm{tod}}\right)∥\sin\left(2\pi T_{bt}^{\mathrm{dow}}\right)∥\cos\left(2\pi T_{bt}^{\mathrm{dow}}\right)∥E_{n}^{n}\right],$$

where $E_{n}^{n}$ is the embedding of node *n*. The anchor pass uses the aligned $X^{a}$, the same temporal covariates and node embeddings, and a unit availability indicator. The support, MAD, and STD channels carry anchor reliability into subsequent modules.

Let $U^{c}$ and $U^{a}$ denote the two encoder-input sequences. The shared GraphGRU encoding is

(8) $$H^{c}=E_{\theta }\left(U^{c},\tilde{A}\right),\;H^{a}=E_{\theta }\left(U^{a},\tilde{A}\right),\;\Delta H=H^{c}-H^{a}.$$

The two passes share the encoder and all its parameters. The paired states therefore occupy a common representation space, and ΔH represents their current–anchor hidden-state deviation. Subsequent modules use both ΔH and |ΔH| to encode its direction and magnitude.

### 4.3. Multi-View Deviation Evidence

ST-FGDIR constructs node-wise evidence from statistical, spatial-neighborhood, hidden-state, and prototype-distribution deviations. The current observation ratio and anchor statistics provide separate reliability channels. Given a MAD floor $\epsilon_{\mathrm{MAD}}>0$, the statistical evidence is

(9) $$\begin{array}{cccc} d_{btn} & =\frac{|X_{btn}^{c}-X_{btn}^{a}|}{\max(S_{btn}^{\mathrm{mad}},\epsilon_{\mathrm{MAD}})}, & \delta_{bn}^{\mathrm{raw}} & =\frac{\sum_{t}M_{btn}^{c}d_{btn}}{\max(\sum_{t}M_{btn}^{c},1)},\\ \rho_{bn}^{\mathrm{obs}} & =\frac{1}{T}\sum_{t}M_{btn}^{c}, & {\bar{s}}_{bn}^{\mathrm{mad}} & =\frac{1}{T}\sum_{t}S_{btn}^{\mathrm{mad}},\\ {\bar{s}}_{bn}^{\mathrm{std}} & =\frac{1}{T}\sum_{t}S_{btn}^{\mathrm{std}}, & {\bar{s}}_{bn}^{\sup} & =\frac{1}{T}\sum_{t}\log\left(1+S_{btn}^{\sup}\right). \end{array}$$

Here, $d_{btn}$ is the point-wise MAD-standardized deviation, $\delta_{bn}^{\mathrm{raw}}$ is its masked temporal aggregation, $\rho_{bn}^{\mathrm{obs}}$ is the current-window observation ratio, and $\left({\bar{s}}_{bn}^{\mathrm{mad}},{\bar{s}}_{bn}^{\mathrm{std}},{\bar{s}}_{bn}^{\sup}\right)$ are the temporally aggregated anchor statistics.

Let $G_{\tilde{A}}\left(Z\right)=\tilde{A}Z$ denote graph mixing over the normalized static graph. The spatial-neighborhood deviation is

(10) $$\delta^{\mathrm{spa}}=\left|\delta^{\mathrm{raw}}-G_{\tilde{A}}\left(\delta^{\mathrm{raw}}\right)\right|.$$

This quantity measures the absolute difference between a node’s own deviation and its graph-mixed neighborhood deviation.

The anchor-conditioned stable path concatenates the paired hidden states, their difference, the spatial context, and statistical reliability, then applies a nonlinear projection:

(11) $$\begin{array}{cc} U^{I}= & [H^{c}∥H^{a}∥\Delta H∥\left|\Delta H\right|∥G_{\tilde{A}}\left(H^{c}\right)∥\rho^{\mathrm{obs}}∥\delta^{\mathrm{raw}}∥\\ & {\bar{s}}^{\mathrm{mad}}∥{\bar{s}}^{\mathrm{std}}∥{\bar{s}}^{\sup}],\;F^{I}=\psi_{I}\left(U^{I}\right). \end{array}$$

The prototype-distribution view uses a learnable codebook $P=\{p_{1},\ldots ,p_{K}\}$, where $p_{k}\in R^{d_{p}}$, *K* is the number of prototypes, and $d_{p}$ is the prototype dimension. A shared linear projection maps the current and anchor states into the prototype space:

(12) $$Q^{c}=W_{q}H^{c}+b_{q},\;Q^{a}=W_{q}H^{a}+b_{q}.$$

For z∈{c,a}, the soft assignment follows scaled dot-product compatibility [36]; the assignment to prototype *m* and the prototype-conditioned representation are

(13) $$\begin{array}{cc} \alpha_{bnm}^{z} & =\frac{\exp\;\left({\left(Q_{bn}^{z}\right)}^{⊤}p_{m}/\sqrt{d_{p}}\right)}{\sum_{j=1}^{K}\exp\;\left({\left(Q_{bn}^{z}\right)}^{⊤}p_{j}/\sqrt{d_{p}}\right)},\\ V_{bn}^{z} & =\sum_{m=1}^{K}\alpha_{bnm}^{z}p_{m}. \end{array}$$

The prototype-assignment logits use the fixed scale $1/\sqrt{d_{p}}$. Assignment uncertainty is represented by normalized entropy with a probability floor $\epsilon_{H}$:

(14) $$\eta_{bn}^{z}=-\frac{1}{\log K}\sum_{m=1}^{K}\alpha_{bnm}^{z}\log\;\left[\max(\alpha_{bnm}^{z},\epsilon_{H})\right].$$

The model then uses the $L_{1}$ distance between prototypes to compute the expected pairwise prototype distance under the current and anchor assignments:

(15) $$D_{mk}={∥p_{m}-p_{k}∥}_{1},\;\delta_{bn}^{\mathrm{proto}}=\sum_{m=1}^{K}\sum_{k=1}^{K}\alpha_{bnm}^{c}D_{mk}\alpha_{bnk}^{a}.$$

Here, $D_{mk}$ is the distance between prototypes *m* and *k*, and $\delta_{bn}^{\mathrm{proto}}$ is the resulting prototype-distribution deviation score.

The complete node-wise multi-view evidence is

(16) $$\begin{array}{cc} F^{D}= & [H^{c}∥H^{a}∥\Delta H∥\left|\Delta H\right|∥V^{c}∥V^{a}∥\delta^{\mathrm{proto}}∥\eta^{c}∥\eta^{a}∥\\ & \delta^{\mathrm{raw}}∥{\bar{s}}^{\mathrm{mad}}∥{\bar{s}}^{\mathrm{std}}∥{\bar{s}}^{\sup}∥\rho^{\mathrm{obs}}∥\delta^{\mathrm{spa}}]. \end{array}$$

The residual decoder and Forecast-Gain Router share $F^{D}$ as their input.

### 4.4. Stable and Deviation-Corrected Forecast Candidates

The stable endpoint decoder combines the anchor-conditioned feature with known future time-of-day covariates. The deviation path projects the multi-view evidence into a deviation latent representation and decodes a horizon-wise residual correction:

(17) $$\begin{array}{cc} {\hat{Y}}^{I} & ={\mathrm{Dec}}_{I}\left(F^{I},T^{y,\mathrm{tod}}\right),\\ Z^{D} & =\psi_{D}\left(F^{D}\right),\\ {\hat{R}}^{D} & ={\mathrm{Dec}}_{D}\left(Z^{D},T^{y,\mathrm{tod}}\right). \end{array}$$

Both decoders expand each node representation along the forecast horizon and concatenate sinusoidal future-time encodings. The stable endpoint and residual correction form ${\hat{Y}}^{D}$ through Equation (2).

### 4.5. Forecast-Gain-Guided Routing

**Figure 3:**
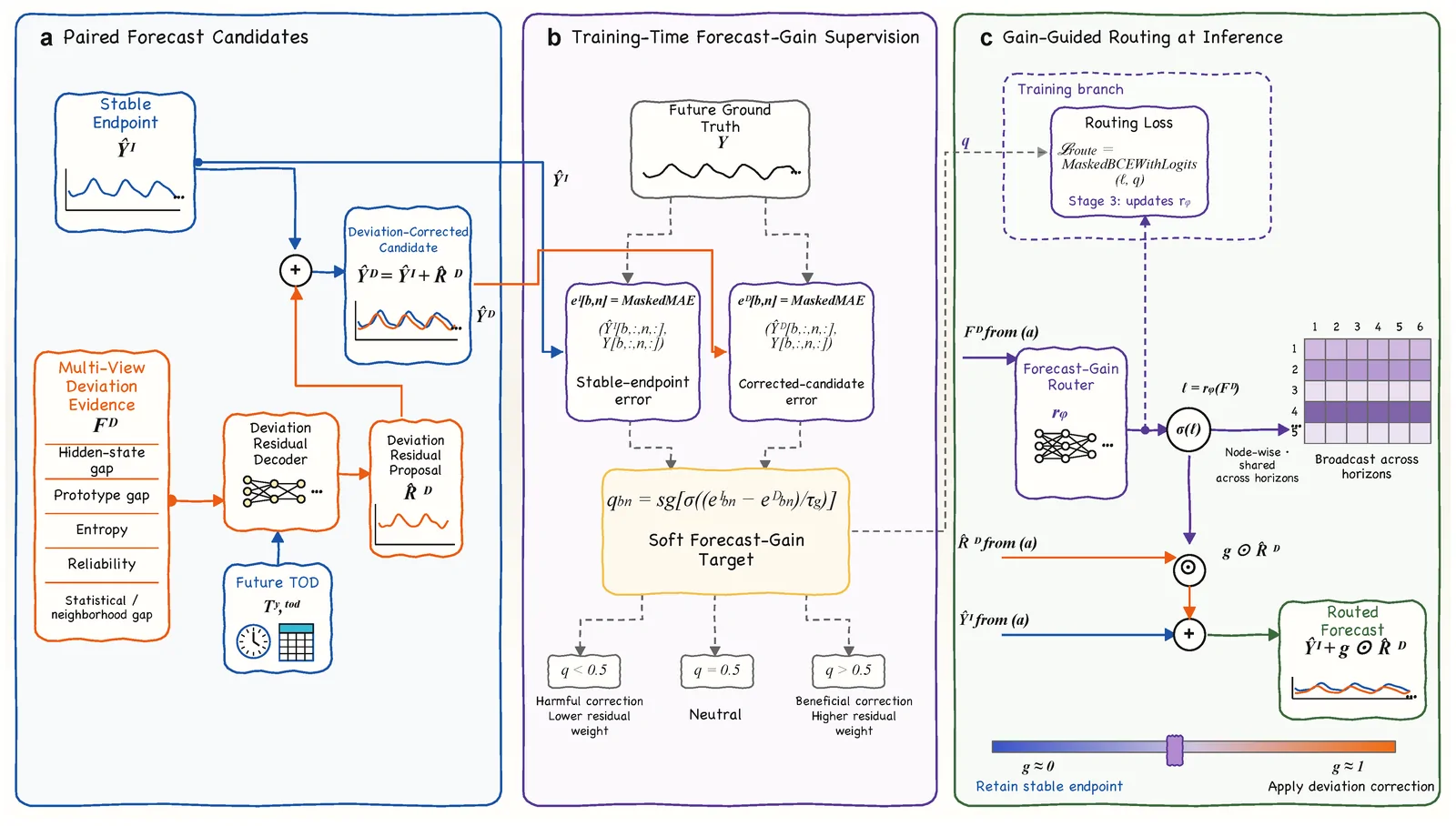
Forecast-gain-guided routing in ST-FGDIR. (**a**) Stable and deviation-corrected forecast candidates. (**b**) Soft forecast-gain targets derived from their masked errors during training. (**c**) The Forecast-Gain Router computes a node-wise, horizon-shared gate from multi-view deviation evidence and controls the residual correction applied to the stable endpoint. Ellipses denote repeated sequence elements or network units; ⊙ and + denote element-wise multiplication and addition, respectively.

Figure 3 shows the training supervision and inference path of the Forecast-Gain Router. Its target is the node-wise forecast gain of the deviation-corrected candidate relative to the stable endpoint. Let $\Omega_{bhn}\in \left\{0,1\right\}$ mark a valid future label, and define $C_{bn}=\sum_{h}\Omega_{bhn}$ and $m_{bn}=I\left[C_{bn}>0\right]$. The node-wise masked errors are

(18) $$\begin{array}{cc} e_{bn}^{I} & =\frac{\sum_{h}\Omega_{bhn}\left|{\hat{Y}}_{bhn}^{I}-Y_{bhn}\right|}{\max(C_{bn},1)},\\ e_{bn}^{D} & =\frac{\sum_{h}\Omega_{bhn}\left|{\hat{Y}}_{bhn}^{D}-Y_{bhn}\right|}{\max(C_{bn},1)}. \end{array}$$

Their difference defines the stop-gradient soft forecast-gain target

(19) $$q_{bn}=\mathrm{sg}\;\left[\sigma \;\left(\frac{e_{bn}^{I}-e_{bn}^{D}}{\tau_{g}}\right)\right],$$

where sg(·) denotes stop-gradient and $\tau_{g}>0$ controls target smoothness. The relations $e_{bn}^{D}<e_{bn}^{I}$, $e_{bn}^{D}>e_{bn}^{I}$, and $e_{bn}^{D}=e_{bn}^{I}$ correspond to $q_{bn}>0.5$, $q_{bn}<0.5$, and $q_{bn}=0.5$, respectively.

The Forecast-Gain Router maps multi-view evidence to node-wise logits and continuous gates:

(20) $$\ell =r_{\phi }\left(F^{D}\right),\;g=\sigma \left(\ell \right),$$

where $\ell \in R^{B\times N\times 1}$. The gate is expanded to $g\in {\left(0,1\right)}^{B\times 1\times N\times 1}$ and broadcast across forecast horizons. The shared gate controls correction strength over the forecasting window, consistent with supervision based on horizon-averaged candidate errors, while the residual prediction remains horizon-specific. Let BCELogit(ℓ,q) denote binary cross-entropy evaluated from a logit. The routing loss is

(21) $$L_{\mathrm{route}}=\frac{\sum_{b,n}m_{bn}\mathrm{BCELogit}\left(\ell_{bn},q_{bn}\right)}{\max\;\left(\sum_{b,n}m_{bn},1\right)}.$$

The mask $m_{bn}$ selects sample–node pairs with valid future labels. For each valid sample–node pair, the routing cross-entropy has logit gradient $g_{bn}-q_{bn}$, providing a corrective signal when the gate disagrees with its soft target. The candidate-generating parameters remain fixed during router training, and stop-gradient prevents this loss from directly updating the target through the candidate errors. During joint fine-tuning, q is recomputed from each clean batch and detached, while the gradient through $F^{D}$ updates the router and the upstream deviation-evidence representation. During inference, the router computes g from $F^{D}$ and produces the final forecast through Equation (3).

### 4.6. Selective Invariance and Four-Stage Optimization

Each prediction loss aggregates future labels through a validity mask Ω. The masked mean absolute error is

(22) $$\mathrm{ML}1\left(\hat{Z},Z\right)=\frac{\sum_{i}\Omega_{i}\left|{\hat{Z}}_{i}-Z_{i}\right|}{\max(\sum_{i}\Omega_{i},1)}.$$

The routed-forecast, stable-endpoint, and deviation-residual losses are

(23) $$\begin{array}{cc} L_{\mathrm{forecast}} & =\mathrm{ML}1(\hat{Y},Y),\\ L_{I} & =\mathrm{ML}1({\hat{Y}}^{I},Y),\\ L_{R} & =\mathrm{ML}1\;\left({\hat{R}}^{D},Y-\mathrm{sg}\left({\hat{Y}}^{I}\right)\right). \end{array}$$

In $L_{R}$, the future labels and the stop-gradient stable endpoint define the residual target. Statistical and prototype deviations are aligned through

(24) $$L_{\mathrm{dev}}=\mathrm{SmoothL}1\;\left(\log\left(1+\delta^{\mathrm{proto}}\right),\log\left[1+\mathrm{sg}\left(\delta^{\mathrm{raw}}\right)\right]\right).$$

Here and in the prototype objective below, SmoothL1 denotes the element-wise smooth $L_{1}$ loss averaged over its tensor arguments. The deviation term uses the stop-gradient statistical deviation as the target scale for the prototype-space deviation.

Following the stop-gradient codebook-learning principle used in vector-quantized representation learning [37], the prototype codebook is optimized by codebook alignment and query commitment, together with prototype diversity. Let ${\bar{p}}_{m}=p_{m}/{∥p_{m}∥}_{2}$. The prototype objective is

(25) $$\begin{array}{cc} L_{\mathrm{code}}= & \mathrm{SmoothL}1\left(V^{c},\mathrm{sg}\left(Q^{c}\right)\right)+\mathrm{SmoothL}1\left(V^{a},\mathrm{sg}\left(Q^{a}\right)\right),\\ L_{\mathrm{com}}= & \mathrm{SmoothL}1\left(Q^{c},\mathrm{sg}\left(V^{c}\right)\right)+\mathrm{SmoothL}1\left(Q^{a},\mathrm{sg}\left(V^{a}\right)\right),\\ L_{\mathrm{div}}= & \frac{1}{K(K-1)}\sum_{m\neq k}{\left[{\bar{p}}_{m}^{⊤}{\bar{p}}_{k}-\gamma \right]}_{+},\\ L_{\mathrm{proto}}= & L_{\mathrm{code}}+\lambda_{\mathrm{com}}L_{\mathrm{com}}+\lambda_{\mathrm{div}}L_{\mathrm{div}}. \end{array}$$

Here, ${\left[x\right]}_{+}=\max\left(x,0\right)$ and γ is the prototype-similarity margin. The codebook term aligns prototype-conditioned representations with stop-gradient query targets, the commitment term aligns queries with stop-gradient prototype-conditioned representations, and the diversity term controls pairwise prototype cosine similarity.

Joint fine-tuning applies label-preserving transformations $T_{k}$ to the clean batch $B^{\left(0\right)}$, giving $B^{\left(k\right)}=T_{k}\left(B^{\left(0\right)}\right)$ for $k=1,\ldots ,K_{e}-1$. The transformations instantiate point missingness, block missingness, and Gaussian noise in the historical inputs while leaving Y and Ω unchanged. The clean environment is indexed by k=0. The environment-specific stable-endpoint error, selective weight, and risk are

(26) $$\begin{array}{cc} e_{bn}^{I,(k)} & =\frac{\sum_{h}\Omega_{bhn}\left|{\hat{Y}}_{bhn}^{I,(k)}-Y_{bhn}\right|}{\max(C_{bn},1)},\\ a_{bn} & =1-\mathrm{sg}(q_{bn}^{\left(0\right)}),\\ R_{k} & =\frac{\sum_{b,n}a_{bn}m_{bn}e_{bn}^{I,(k)}}{\max\;\left(\sum_{b,n}a_{bn}m_{bn},10^{-6}\right)}. \end{array}$$

The clean-environment target $q_{bn}^{\left(0\right)}$ defines the selective weight $a_{bn}$, which emphasizes sample–node pairs whose routing targets favor the stable endpoint. Following the risk-extrapolation principle of jointly controlling average risk and cross-environment risk variation [11], ST-FGDIR defines its selective counterpart as

(27) $$\begin{array}{cc} \bar{R} & =\frac{1}{K_{e}}\sum_{k=0}^{K_{e}-1}R_{k},\\ {\mathrm{Var}}_{\mathrm{pop}}\left(R\right) & =\frac{1}{K_{e}}\sum_{k=0}^{K_{e}-1}{\left(R_{k}-\bar{R}\right)}^{2},\\ L_{\mathrm{sREx}} & =\bar{R}+\lambda_{\mathrm{var}}{\mathrm{Var}}_{\mathrm{pop}}\left(R\right). \end{array}$$

For $K_{e}=1$, the population variance equals zero and $L_{\mathrm{sREx}}=R_{0}$.

The false-activation loss measures positive gate increments in perturbed environments relative to the clean environment:

(28) $$L_{\mathrm{fa}}=\left\{\begin{array}{cc} \frac{1}{K_{e}-1}\sum_{k=1}^{K_{e}-1}\frac{1}{BN}\sum_{b,n}{\left[g_{bn}^{\left(k\right)}-\mathrm{sg}\left(g_{bn}^{\left(0\right)}\right)\right]}_{+}, & K_{e}>1,\\ 0, & K_{e}=1. \end{array}\right.$$

The stop-gradient clean-environment gate provides a fixed reference, and gradients propagate through the gates and upstream evidence representations of perturbed environments.

The joint fine-tuning objective is

(29) $$\begin{array}{cc} L_{\mathrm{joint}}= & \lambda_{f}L_{\mathrm{forecast}}+\lambda_{I}L_{I}+\lambda_{R}L_{R}+\lambda_{g}L_{\mathrm{route}}\\ & +\lambda_{\mathrm{sREx}}L_{\mathrm{sREx}}+\lambda_{\mathrm{fa}}L_{\mathrm{fa}}+\lambda_{p}L_{\mathrm{proto}}+\lambda_{d}L_{\mathrm{dev}}. \end{array}$$

The forecast, stable-endpoint, residual, routing, prototype, and deviation terms use the clean environment. The selective risk-extrapolation and false-activation terms jointly use the clean and perturbed environments.

The four-stage objective is

(30) $$L^{\left(s\right)}=\left\{\begin{array}{cc} \lambda_{I}L_{I}, & s=1,\\ \lambda_{R}L_{R}+\lambda_{p}L_{\mathrm{proto}}+\lambda_{d}L_{\mathrm{dev}}, & s=2,\\ \lambda_{g}L_{\mathrm{route}}, & s=3,\\ L_{\mathrm{joint}}, & s=4. \end{array}\right.$$

Here, s∈{1,2,3,4} is the training-stage index. Stage 1 performs stable-path warm-up. The trainable modules are the node embeddings, shared encoder, stable-feature projector, and stable endpoint decoder; the prototype module, deviation feature projector, deviation residual decoder, and Forecast-Gain Router remain fixed. Stage 2 performs deviation-residual and prototype warm-up. The trainable modules are the prototype module, deviation feature projector, and deviation residual decoder; the node embeddings, shared encoder, stable-feature projector, stable endpoint decoder, and Forecast-Gain Router remain fixed. Stage 3 trains $r_{\phi }$ with $L_{\mathrm{route}}$ while the remaining model parameters stay fixed. Stage 4 performs multi-environment joint fine-tuning with all model parameters trainable.

## 5. Experiments

### 5.1. Experimental Setup

**Datasets.** We evaluated ST-FGDIR on four public traffic forecasting benchmarks: METR-LA, PEMS-BAY, PEMS04, and PEMS08. METR-LA and PEMS-BAY contain traffic-speed measurements, whereas PEMS04 and PEMS08 contain traffic-flow measurements. All four datasets were sampled at 5 min intervals. Their statistics are summarized in Table 1.

METR-LA and PEMS-BAY were chronologically divided into training, validation, and test sets using a ratio of 70%/10%/20%, whereas PEMS04 and PEMS08 were divided using a ratio of 60%/20%/20%. Each sample contained 12 historical time steps and 12 prediction steps, corresponding to forecasting the next 60 min from the preceding 60 min. Traffic measurements were normalized using statistics estimated from the training set. The periodic anchor table and its associated MAD, STD, and support statistics were fitted over the complete training period and then fixed for all training, validation, and test samples. Keeping these estimates fixed provides a common periodic reference for evaluating residual routing under the offline protocol.

**Evaluation metrics.** Forecasting performance was evaluated using mean absolute error (MAE), root mean squared error (RMSE), and mean absolute percentage error (MAPE) over valid test measurements. Lower values indicate better forecasting performance.

**Implementation settings.** ST-FGDIR was optimized using Adam with a batch size of 16. The learning rate was set to 10^−3^ during the first three training stages and reduced to 3 × 10^−4^ during joint fine-tuning. The GraphGRU hidden-state dimension was set to 16 for METR-LA and PEMS08 and 12 for PEMS-BAY and PEMS04. The prototype bank contained K=8 prototypes with dimension $d_{p}=8$, and the forecast-gain smoothing coefficient was set to $\tau_{g}=0.25$ for endpoint-error differences on the normalized scale. The four training stages were each conducted for one epoch on METR-LA, PEMS-BAY, and PEMS04. For PEMS08, the joint fine-tuning stage was extended to two epochs. All experiments were performed on an NVIDIA GeForce RTX 4090 GPU.

Table 2 lists the loss coefficients and numerical constants used for PEMS04. During joint fine-tuning, each batch contained one clean view and two perturbed views generated independently from it, cycling through point/block, block/noise, and noise/point pairs. Point missingness independently masked 15% of valid observations. Block missingness masked two consecutive steps per sample–node pair, with independently and uniformly sampled valid start positions. Gaussian noise had a standard deviation equal to 15% of each node’s historical-window standard deviation. Newly masked normalized inputs and their observation indicators were set to zero, while future targets remained unchanged.

### 5.2. Overall Forecasting Performance

Table 3 compares ST-FGDIR with ten representative traffic forecasting models. For METR-LA and PEMS-BAY, the results are reported at 15, 30, and 60 min, corresponding to the third, sixth, and twelfth steps of the same 12-step forecasting task. For PEMS04 and PEMS08, each metric is averaged over all 12 forecasting steps. In Table 3, results from DCRNN through ST-SSDL are taken from the comparison reported by Gao et al. [9], and TRL-DAG results are taken from Chen et al. [38]. These studies use the same dataset split ratios, input and prediction lengths, and horizon-wise metric reporting as this study, while preprocessing and metric implementations remain those of the source studies. ST-FGDIR results are obtained using the protocol described in Section 5.1. TRL-DAG [38] combines temporal representation learning with dynamic graph modeling and adversarial training.

Among the results compiled in Table 3, ST-FGDIR achieves the lowest reported error in 21 of the 24 evaluation entries and the second-lowest in the remaining three. On METR-LA, ST-FGDIR ranked first among the results in Table 3 for all nine combinations of forecasting horizon and evaluation metric. Its MAE/RMSE/MAPE values were 2.58/5.01/6.42% at 15 min, 2.95/6.01/7.84% at 30 min, and 3.24/7.03/9.30% at 60 min. Relative to the published ST-SSDL results, the corresponding error reductions ranged from 0.20% to 3.86%, with the largest reduction obtained for the 60 min MAE, which decreased from 3.37 to 3.24.

On PEMS-BAY, ST-FGDIR obtained the best reported value in seven of the nine evaluation entries in Table 3. At 15 min, it achieved an MAE of 1.22, an RMSE of 2.59, and a MAPE of 2.54%; at 30 min, the corresponding values were 1.52, 3.56, and 3.41%. These six results were all lower than the corresponding published baseline values. At 60 min, ST-FGDIR achieved the lowest RMSE of 4.32, while its MAE of 1.89 was tied with MegaCRN for second place and its MAPE of 4.39 ranked second.

For the traffic-flow datasets, ST-FGDIR achieved an MAE of 17.99, an RMSE of 29.14, and a MAPE of 12.13% on PEMS04. Its MAE and RMSE were the lowest among the methods listed in Table 3, while its MAPE ranked second to the 12.04% obtained by STID. On PEMS08, ST-FGDIR achieved the lowest reported values in Table 3 for all three metrics, with an MAE of 13.82, an RMSE of 23.04, and a MAPE of 9.06%. Compared with the published ST-SSDL results, ST-FGDIR produced lower errors for all six metrics on PEMS04 and PEMS08, with relative reductions ranging from 0.26% to 1.86%.

Table 4 summarizes the computational costs on PEMS04 using a single NVIDIA RTX 4090 GPU, FP32, and a batch size of 16. Training times cover four epochs, excluding preprocessing, validation, and checkpoint saving. Inference times measure model forward passes after warm-up and GPU synchronization. Memory denotes peak allocated GPU memory during training.

ST-FGDIR uses fewer parameters and requires less training and inference time than ST-SSDL, at the cost of higher peak memory usage. STID remains faster and uses less memory.

### 5.3. Ablation Study

To examine the roles of the model mechanisms and training strategies in ST-FGDIR, we constructed nine ablation variants. The model-mechanism group comprised Stable Endpoint Only (g=0), Always-On Residual (g=1), w/o Periodic Anchor, w/o Statistical–Spatial Evidence, and w/o Prototype-Distribution Evidence. The training-objective and optimization group removed forecast-gain supervision, selective risk extrapolation (sREx), the false-activation loss, or the four-stage optimization schedule. The w/o Periodic Anchor variant excludes the training-derived periodic reference. The w/o Forecast-Gain Supervision variant removes explicit supervision of the routing gate using the candidate-error-based gain target. Table 5 reports MAE and RMSE computed over the complete 12-step forecasting window. All variants used the same data splits, total number of training epochs, and evaluation protocol. The single-stage variant jointly optimized all model parameters under the same training budget.

Full ST-FGDIR achieved the lowest error in all eight evaluation entries. Relative to Always-On Residual, its MAE reductions were 0.69%, 0.66%, 0.72%, and 0.67% on METR-LA, PEMS-BAY, PEMS04, and PEMS08, respectively. These aggregate improvements are modest. The gate-group analysis in Section 5.4 examines how routing affects predictions under different correction utilities.

Among the model-mechanism variants, removing the periodic anchor produced the largest degradation. The corresponding MAE/RMSE values increased to 2.980/6.134, 1.554/3.565, 18.623/30.081, and 14.322/23.791 on METR-LA, PEMS-BAY, PEMS04, and PEMS08, respectively. Removing statistical–spatial evidence increased the PEMS04 and PEMS08 results from 17.990/29.140 and 13.820/23.040 to 18.332/29.650 and 14.072/23.464, respectively. Removing prototype-distribution evidence also increased both metrics on all four datasets. Removing each evidence source increased both MAE and RMSE across the four datasets.

The training-objective and optimization variants exhibited a consistent pattern. On PEMS04, removing forecast-gain supervision increased MAE/RMSE from 17.990/29.140 to 18.280/29.577, while removing sREx increased them to 18.215/29.493. Removing the false-activation loss produced smaller but consistent increases across all four datasets. Under the matched training budget, the single-stage variant produced the largest degradation among the optimization variants, yielding 2.999/6.184, 1.563/3.596, 18.746/30.224, and 14.405/23.957 on the four datasets.

### 5.4. Forecast-Gain Router Analysis

To examine the relationship between the routing gate and the utility of residual correction, we divided the valid test sample–node pairs in each dataset into ten equal-frequency groups, D1–D10, according to the inference gate *g* from low to high. Using the future observations, we computed the node-wise masked errors $e^{I}$ and $e^{D}$ of the stable endpoint and deviation-corrected candidate, respectively, and defined the realized forecast gain as $\Delta e=e^{I}-e^{D}$. Accordingly, Δe<0 indicates a lower error for the stable endpoint, whereas Δe>0 indicates that the deviation-corrected candidate improves the forecast.

**Figure 4:**
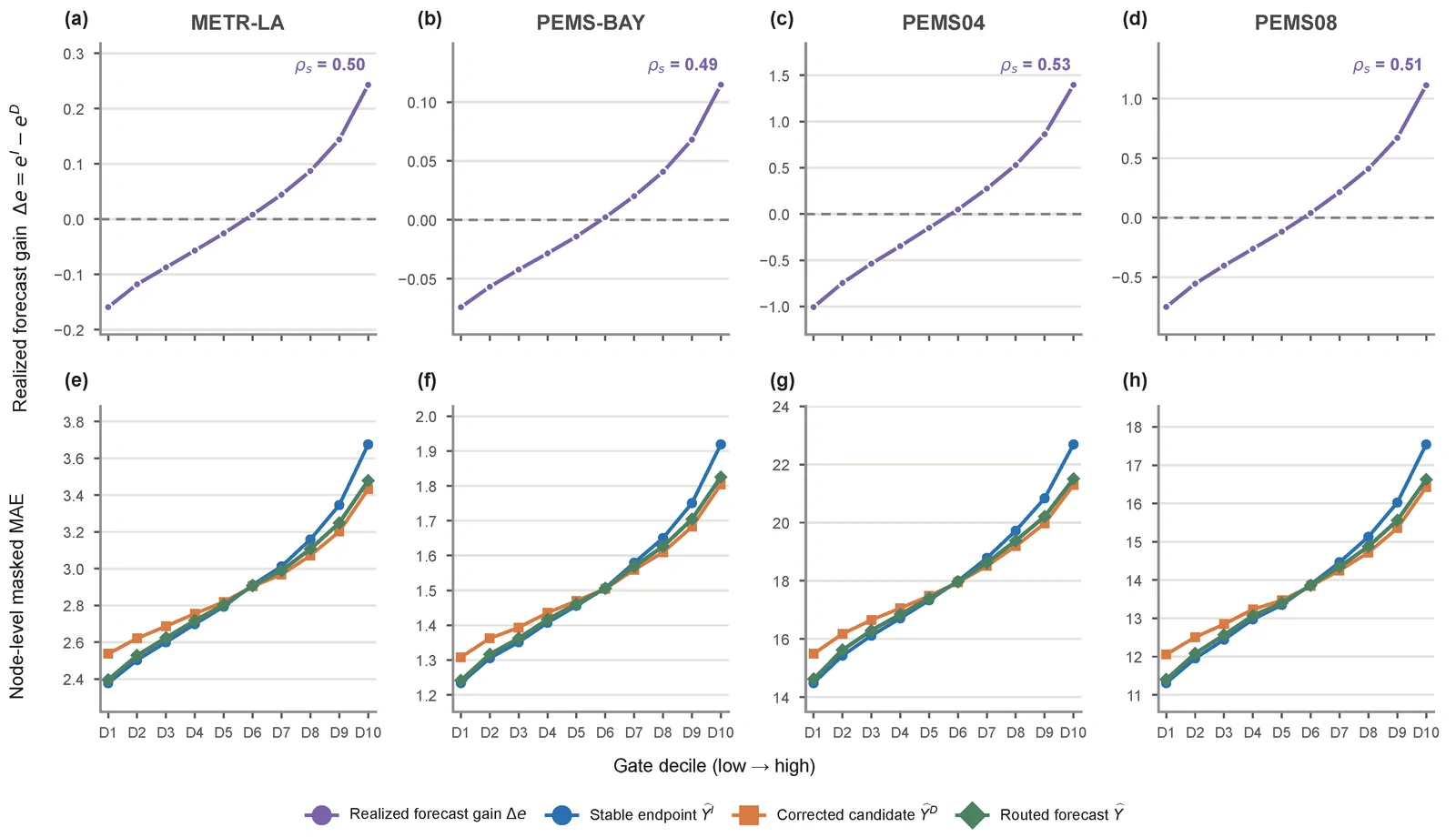
Forecast-gain alignment of the router. (**a**–**d**) Bin-averaged realized forecast gain on METR-LA, PEMS-BAY, PEMS04, and PEMS08. (**e**–**h**) Node-level masked MAE of the stable endpoint, deviation-corrected candidate, and routed forecast within the same gate groups. D1 and D10 denote the lowest and highest gate deciles, respectively. The horizontal dashed lines in (**a**–**d**) indicate zero forecast gain.

As shown in Figure 4a–d, the bin-averaged forecast gain increased with the gate decile on all four datasets. It was negative in the low-gate groups, crossed zero near D6, and became positive in the high-gate groups. The Spearman rank correlations, computed from the unbinned valid sample–node pairs, were 0.50, 0.49, 0.53, and 0.51 on METR-LA, PEMS-BAY, PEMS04, and PEMS08, respectively. These values indicate a consistent, moderate positive rank association between the inference gate and realized correction utility across the four datasets.

Figure 4e–h further compares the three forecasts within the same gate groups. The stable endpoint had the lower MAE in the low-gate groups, the candidate errors converged near D6, and the deviation-corrected candidate had the lower MAE in the high-gate groups. The routed forecast tracked the lower-error candidate more closely in the low- and high-gate regions. The benefit over complete residual injection is evident in the low-gate groups, where the corrected candidate has higher error than the stable endpoint and routing suppresses its contribution. In the high-gate groups, the routed forecast approaches the lower-error corrected candidate. These patterns indicate that the effect of routing depends on the utility of the proposed correction.

As shown in Table 6, the gate–gain correlations on PEMS04 remain positive across all 12 forecasting horizons (Spearman *ρ* = 0.5239–0.5248), and the routed forecast achieves lower MAE than both the stable endpoint and the deviation-corrected candidate at every horizon. These results support the use of the shared gate under the evaluated setting.

### 5.5. Qualitative Routing Cases

**Figure 5:**
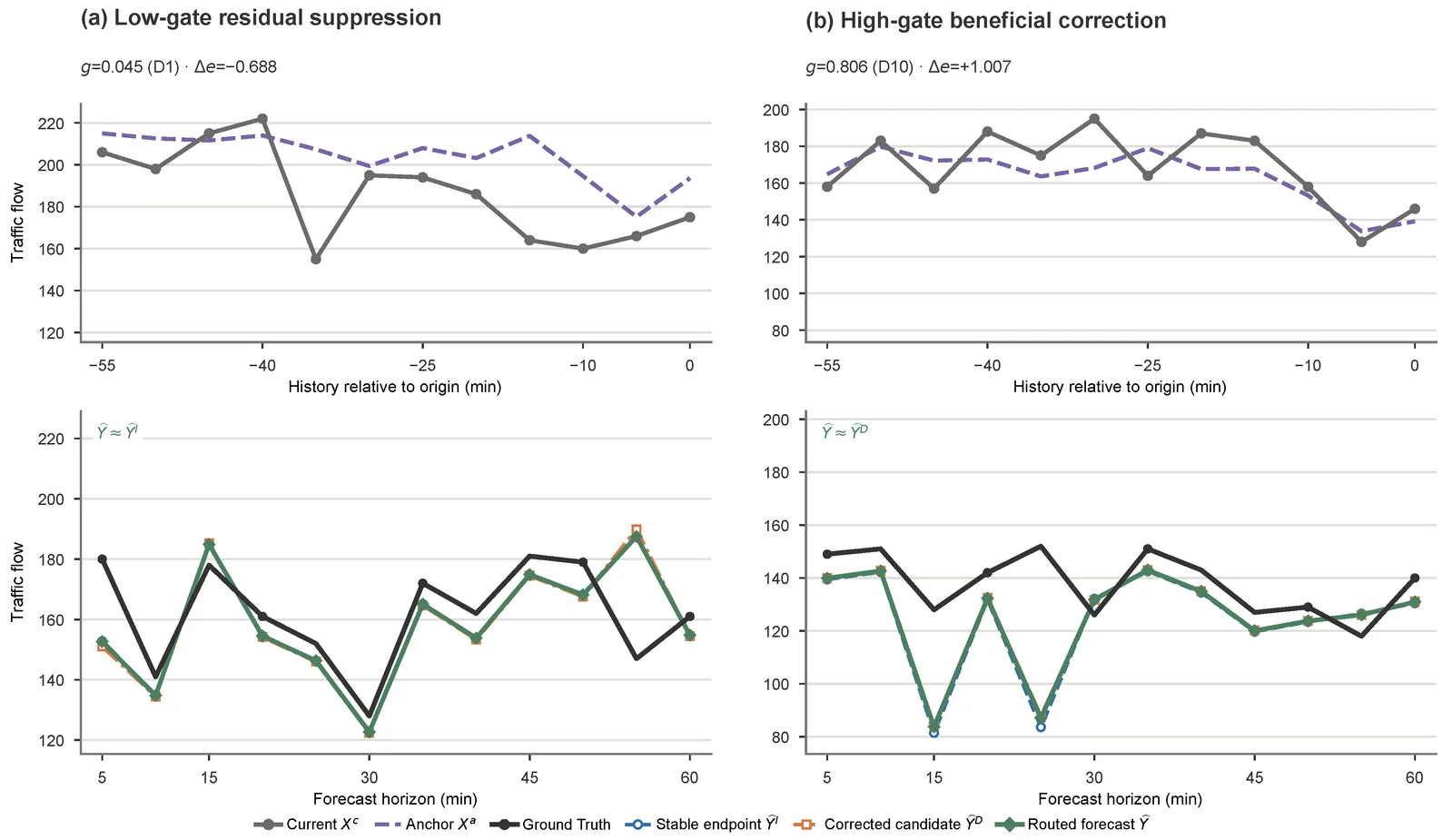
Illustrative gain-aligned routing cases on PEMS08. The upper panels compare the current historical sequence with its periodic anchor, while the lower panels show the future ground truth, stable endpoint, deviation-corrected candidate, and routed forecast. (**a**) Low-gate residual suppression with g=0.045 and Δe=−0.688. (**b**) High-gate beneficial correction with g=0.806 and Δe=1.007. Here, $\Delta e=e^{I}-e^{D}$.

Figure 4 shows that the realized forecast gain $\Delta e=e^{I}-e^{D}$ increased across gate deciles at the aggregate level. Figure 5 complements this relationship with two illustrative, gain-aligned PEMS08 cases from the lowest gate decile D1 and the highest gate decile D10. The cases were selected from sample–node pairs with Δe<0 and Δe>0, respectively, by choosing the pair closest to the median gain in each eligible set. The upper panels compare the current historical sequence with its periodic anchor, while the lower panels show the future ground truth, stable endpoint, deviation-corrected candidate, and final routed forecast.

At the trajectory level, Equation (3) makes the gate a continuous residual-scaling coefficient. In Figure 5a, the router produced g=0.045, and the realized gain was Δe=−0.688, indicating a higher error after applying the complete residual correction. The routed forecast therefore comprised 95.5% of the stable endpoint and 4.5% of the deviation-corrected candidate, and its trajectory remained close to the stable endpoint. The multi-view evidence was associated with low correction utility, so the router restricted residual injection.

Figure 5b shows the complementary behavior. Here, g=0.806 and Δe=1.007, indicating that the deviation-corrected candidate had the lower error. The routed forecast retained 80.6% of the complete residual correction and consequently approached the corrected candidate. The evidence pattern was associated with high correction utility, allowing the correction represented by the residual branch to contribute more strongly to the final prediction.

The two trajectories instantiate the aggregate gate–gain association in Figure 4: the low-gate case remains near the stable endpoint when the complete correction is harmful, whereas the high-gate case moves toward the deviation-corrected candidate when the correction is beneficial.

### 5.6. Robustness Under Controlled Input Perturbations

**Figure 6:**
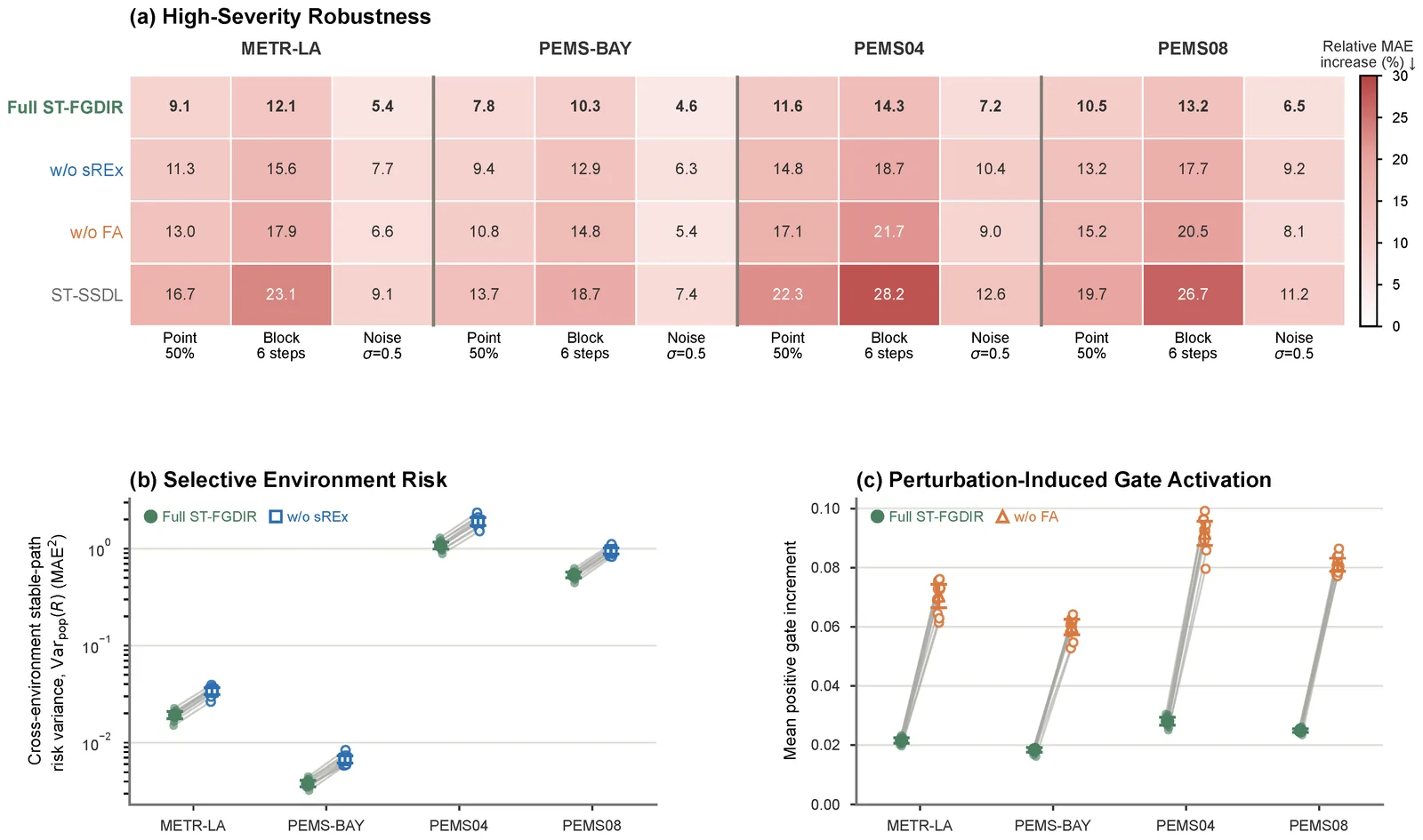
Robustness under controlled input perturbations. For each dataset–perturbation condition, all models are evaluated using fixed, trained checkpoints and the same ten paired corruption realizations, from which the 95% confidence intervals are calculated. (**a**) Relative MAE increase under 50% point missingness, a six-step missing block, and Gaussian noise with σ=0.5, calculated as $100\left({\mathrm{MAE}}_{\mathrm{perturbed}}-{\mathrm{MAE}}_{\mathrm{clean}}\right)/{\mathrm{MAE}}_{\mathrm{clean}}$. Each cell shows the mean over the paired realizations. (**b**) Population variance of the cross-environment stable-path risk for full ST-FGDIR and the variant without sREx; the vertical axis uses a logarithmic scale. (**c**) Mean positive gate increment for full ST-FGDIR and the variant without the false-activation loss. Gray lines connect results sharing the same corruption seed. Larger markers denote the mean, and error bars denote the two-sided 95% Student-*t* confidence interval $\bar{x}\pm t_{0.975,9}s/\sqrt{10}$. Lower values indicate better performance in all panels.

To evaluate predictive stability when historical observations were corrupted, we applied three high-severity perturbations to the 12-step historical inputs: 50% point missingness, a contiguous missing block of six time steps, and Gaussian noise with σ=0.5 in the normalized input space. The future targets remained unchanged. For each dataset–perturbation condition, every checkpoint was evaluated on the same ten perturbation realizations, paired by corruption seed. Two-sided 95% confidence intervals across these realizations were calculated as $\bar{x}\pm t_{0.975,9}s/\sqrt{10}$, where *s* is the sample standard deviation of the ten realization-level statistics. We compared full ST-FGDIR with variants without selective risk extrapolation (sREx) or the false-activation loss, as well as with ST-SSDL. Figure 6a reports the MAE increase of each model relative to its own clean-input performance, while Figure 6b,c directly examine the quantities controlled by sREx and the false-activation loss.

Among the models in Figure 6a, full ST-FGDIR produced the smallest relative MAE increase in all 12 dataset–perturbation combinations. Averaged across these conditions, its increase was 9.38%, compared with 12.27% without sREx, 13.34% without the false-activation loss, and 17.45% for ST-SSDL. Block missingness produced the largest degradation, plausibly because it removes a contiguous segment of short-term dynamics. Point missingness retained interspersed observations, whereas Gaussian corruption retained the complete temporal positions and the observation mask.

Figure 6b isolates the effect of sREx on the stable path. Relative to the variant without sREx, the full model reduced the population variance of the cross-environment stable-path risk by 43.3–43.6% across the four datasets. The clean-environment weighting emphasizes sample–node pairs that favor the stable endpoint, and the lower risk variation coincided with smaller relative MAE increases in Figure 6a.

Figure 6c examines perturbation-induced changes in the routing decision. Across the four datasets, full ST-FGDIR reduced the mean positive gate increment by 69.3–69.4% relative to the variant without the false-activation loss. Because this loss penalizes positive increments relative to the clean-input gate, the result directly shows stronger restraint of perturbation-induced residual weighting and coincides with smaller end-to-end MAE degradation in Figure 6a.

Table 7 compares ST-FGDIR with the invariant-learning baseline DIST [35] on PEMS04 under matched perturbations. DIST achieved lower MAE under clean inputs and Gaussian noise, but ST-FGDIR achieved lower MAE and smaller relative increases under point and block missingness.

### 5.7. Training Dynamics

**Figure 7:**
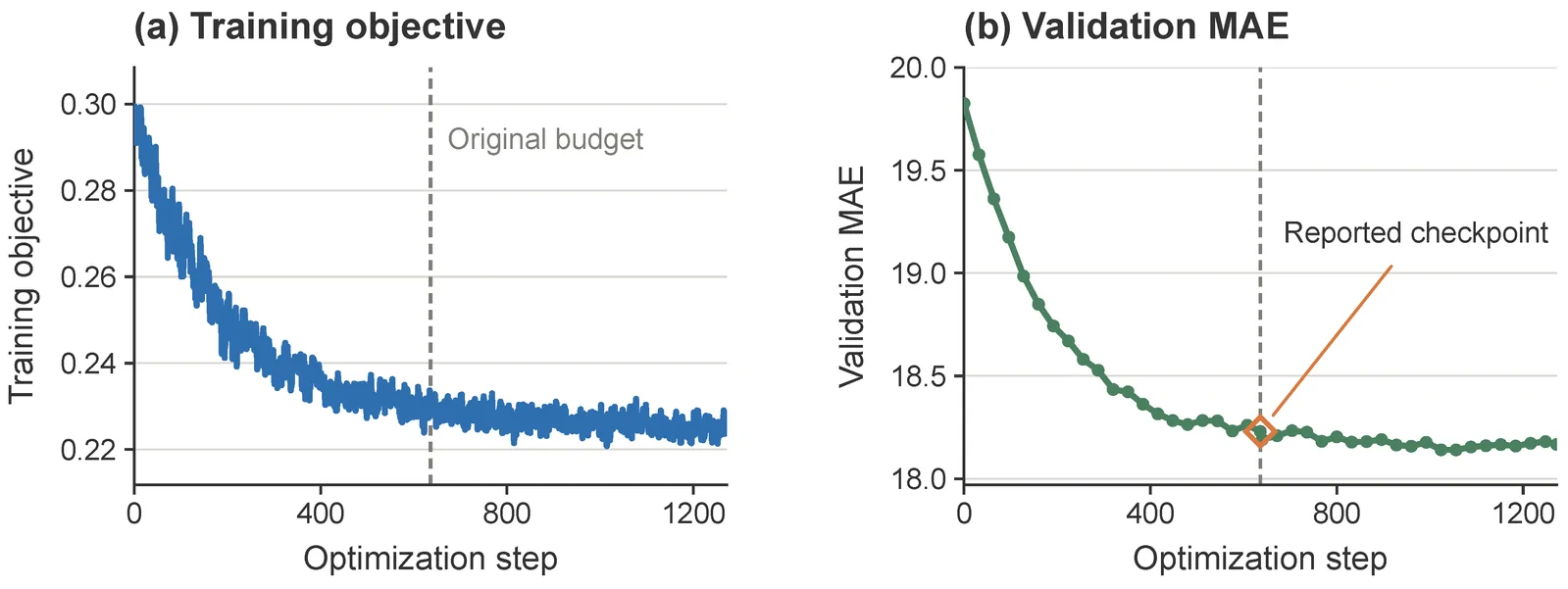
Training dynamics during joint fine-tuning on PEMS04 with 12-step input and output windows. (**a**) Training objective. (**b**) Validation MAE. Dashed lines mark the end of the original training budget, and the orange diamond identifies the checkpoint used for the reported results. Curves beyond the dashed lines show extended training.

Joint fine-tuning optimizes all model parameters following the staged warm-up of the stable path, deviation-residual and prototype modules, and router. Figure 7 shows the training objective and validation MAE on PEMS04, including the trajectory beyond the original training budget. The training objective decreased rapidly during the initial updates and subsequently declined more gradually. Validation MAE exhibited a similar pattern and approached a plateau near the original stopping point. Extended training yielded a small additional reduction in validation MAE, accompanied by minor fluctuations. On PEMS04, most of the validation-error reduction observed over this trajectory occurred within the original training budget. Whether the same training budget is sufficient for METR-LA, PEMS-BAY, and PEMS08 remains to be evaluated.

## 6. Discussion and Conclusions

This study formulates deviation-aware traffic forecasting as a correction-utility problem. ST-FGDIR constructs an anchor-conditioned stable endpoint and a deviation-corrected candidate, then uses their signed error difference to supervise continuous node-wise residual routing. The framework assigns distinct roles to its two signals: multi-view deviation evidence characterizes a current–anchor departure, whereas forecast gain determines how strongly the corresponding residual enters the prediction. This separation positions forecast gain as the control variable connecting deviation representation to selective stabilization.

Three findings characterize the resulting behavior. First, ST-FGDIR achieved competitive forecasting performance relative to the published baseline results compiled in Table 3. Second, the full model outperformed both fixed-gate variants on all four datasets, and the inference gate showed moderate positive rank associations of 0.49–0.53 with realized correction gain. The fixed-gate ablations and gate–gain analysis jointly support conditional residual routing over uniform correction. The crossover in candidate errors near the middle gate deciles further shows that the preferred candidate changes across routing regions. Third, ST-FGDIR showed smaller relative MAE increases than ST-SSDL and the two ablated variants across the 12 controlled dataset–perturbation conditions, with an average increase of 9.38%. Direct measurements further showed lower cross-environment stable-path risk variation with sREx and smaller perturbation-induced gate increments with false-activation regularization. These quantities provide mechanism-specific evidence beyond aggregate robustness error. Together, the findings connect forecasting accuracy, correction utility, and perturbation stability within the same routing framework.

The evaluation covers four public traffic-sensor benchmarks sampled at five-minute intervals and three controlled perturbation types. Long-term changes in recurring traffic patterns may reduce the representativeness of the fixed training-derived anchor, and online anchor updating is outside the present evaluation. Future work will examine online or periodic re-estimation using only observations available before each forecast, alongside naturally occurring sensor faults, traffic events, topology changes, cross-network transfer, and horizon-aware routing. Overall, ST-FGDIR coordinates an anchor-conditioned stable endpoint with adaptive deviation correction under clean and perturbed inputs.

## Figures and Tables

**Table 1:** Statistics of the four traffic benchmark datasets.

Dataset	#Nodes	#Time Steps	#Edges
METR-LA	207	34,272	1515
PEMS-BAY	325	52,116	2369
PEMS04	307	16,992	680
PEMS08	170	17,856	548

**Table 2:** Loss coefficients and numerical constants for PEMS04.

Parameter	Value	Parameter	Value
$\lambda_{f}$	1.00	$\lambda_{I}$, $\lambda_{R}$	0.20
$\lambda_{g}$, $\lambda_{\mathrm{var}}$	0.10	$\lambda_{\mathrm{sREx}}$, $\lambda_{\mathrm{div}}$	0.05
$\lambda_{\mathrm{fa}}$	0.02	$\lambda_{p}$, $\lambda_{d}$	0.01
$\lambda_{\mathrm{com}}$	0.25	γ	0.20
Risk denominator floor	10^−6^	$\epsilon_{\mathrm{MAD}}$	10^−3^
$\epsilon_{H}$	10^−8^		

**Table 3:** Forecasting performance on four traffic benchmark datasets. Results on METR-LA and PEMS-BAY are reported at 15, 30, and 60 min, whereas results on PEMS04 and PEMS08 are averaged over all 12 forecasting steps. Lower values indicate better performance. Bold and underlined values denote the best and second-best reported values in this comparison, respectively.

Dataset	Horizon	Metric	DCRNN [1]	STGCN [15]	GWNet [2]	MTGNN [16]	AGCRN [17]	STID [18]	MegaCRN [3]	STDN [7]	ST-SSDL [9]	TRL-DAG [38]	ST-FGDIR
METR-LA	Step 3 15 min	MAE	2.67	2.75	2.69	2.69	2.85	2.82	2.62	2.83	2.60	2.73	**2.58**
		RMSE	5.16	5.29	5.15	5.16	5.53	5.53	5.04	5.84	5.02	5.32	**5.01**
		MAPE (%)	6.86	7.10	6.99	6.89	7.63	7.75	6.68	7.78	6.62	7.27	**6.42**
	Step 6 30 min	MAE	3.12	3.15	3.08	3.05	3.20	3.19	3.01	3.21	2.96	3.16	**2.95**
		RMSE	6.27	6.35	6.20	6.13	6.52	6.57	6.13	6.92	6.04	6.41	**6.01**
		MAPE (%)	8.42	8.62	8.47	8.16	9.00	9.39	8.18	9.40	7.92	8.68	**7.84**
	Step 12 60 min	MAE	3.54	3.60	3.51	3.47	3.59	3.55	3.48	3.57	3.37	3.55	**3.24**
		RMSE	7.47	7.43	7.28	7.21	7.45	7.55	7.31	7.80	7.17	7.37	**7.03**
		MAPE (%)	10.32	10.35	9.96	9.70	10.47	10.95	9.98	10.98	9.48	10.28	**9.30**
PEMS-BAY	Step 3 15 min	MAE	1.31	1.36	1.30	1.33	1.35	1.31	1.28	1.36	1.26	1.29	**1.22**
		RMSE	2.76	2.88	2.73	2.80	2.88	2.79	2.71	2.96	2.65	2.75	**2.59**
		MAPE (%)	2.73	2.86	2.71	2.81	2.91	2.78	2.67	2.91	2.60	2.78	**2.54**
	Step 6 30 min	MAE	1.65	1.70	1.63	1.66	1.67	1.64	1.60	1.69	1.57	1.61	**1.52**
		RMSE	3.75	3.84	3.73	3.77	3.82	3.73	3.68	3.94	3.59	3.70	**3.56**
		MAPE (%)	3.71	3.79	3.73	3.75	3.81	3.73	3.59	3.81	3.49	3.66	**3.41**
	Step 12 60 min	MAE	1.97	2.02	1.99	1.95	1.94	1.91	1.89	1.92	**1.86**	1.92	1.89
		RMSE	4.60	4.63	4.60	4.50	4.50	4.42	4.45	4.58	4.34	4.50	**4.32**
		MAPE (%)	4.68	4.72	4.71	4.62	4.55	4.55	4.48	4.54	**4.35**	4.56	4.39
PEMS04	Average	MAE	19.63	19.57	18.53	19.17	19.38	18.38	18.69	18.40	18.08	19.05	**17.99**
		RMSE	31.26	31.38	29.92	31.70	31.25	29.95	30.54	30.22	29.64	31.29	**29.14**
		MAPE (%)	13.59	13.44	12.89	13.37	13.40	**12.04**	12.71	12.50	12.36	12.30	12.13
PEMS08	Average	MAE	15.22	16.08	14.40	15.18	15.32	14.21	14.83	14.25	13.86	15.14	**13.82**
		RMSE	24.17	25.39	23.39	24.24	24.41	23.28	23.92	24.39	23.10	24.42	**23.04**
		MAPE (%)	10.21	10.60	9.21	10.20	10.03	9.27	9.64	10.67	9.17	9.63	**9.06**

**Table 4:** Computational costs on PEMS04. Time and memory values are approximate; K denotes 1000 parameters.

Model	Parameters (K)	Training Time, 4 Epochs (s)	Inference Time (ms/batch)	Peak Training Memory (GiB)
STID	121	20	1.1	0.3
ST-SSDL	182	288	52	1.5
ST-FGDIR	8.983	220	47	2.3

**Table 5:** Ablation results on four traffic forecasting datasets. All metrics are computed over the complete 12-step forecasting window. Lower values indicate better performance, and the best results are shown in bold.

Variant	METR-LA		PEMS-BAY		PEMS04		PEMS08	
	MAE	RMSE	MAE	RMSE	MAE	RMSE	MAE	RMSE
Model Mechanisms								
Stable Endpoint Only (g=0)	2.908	6.002	1.516	3.487	18.154	29.382	13.949	23.247
Always-On Residual (g=1)	2.900	5.994	1.513	3.481	18.121	29.356	13.913	23.266
w/o Periodic Anchor	2.980	6.134	1.554	3.565	18.623	30.081	14.322	23.791
w/o Statistical–Spatial Evidence	2.931	6.055	1.531	3.520	18.332	29.650	14.072	23.464
w/o Prototype-Distribution Evidence	2.910	6.011	1.516	3.492	18.166	29.431	13.958	23.284
Training Objectives and Optimization								
w/o Forecast-Gain Supervision	2.923	6.041	1.525	3.508	18.280	29.577	14.033	23.404
w/o Selective REx	2.914	6.024	1.520	3.499	18.215	29.493	13.991	23.344
w/o False-Activation Loss	2.889	5.972	1.508	3.466	18.046	29.227	13.864	23.116
w/o Four-Stage Optimization	2.999	6.184	1.563	3.596	18.746	30.224	14.405	23.957
**Full ST-FGDIR**	**2.880**	**5.950**	**1.503**	**3.455**	**17.990**	**29.140**	**13.820**	**23.040**

**Table 6:** Horizon-wise routing analysis on PEMS04. Spearman’s ρ measures the association between the gate and the reduction in absolute error from the stable endpoint to the deviation-corrected candidate, computed over valid test sample–node pairs at each horizon.

Horizon	Spearman ρ	Stable Endpoint MAE	Deviation-Corrected Candidate MAE	Routed Forecast MAE
1	0.5248	15.249	15.222	15.112
2	0.5244	15.975	15.946	15.831
3	0.5244	16.520	16.490	16.371
4	0.5242	17.064	17.034	16.911
5	0.5242	17.610	17.577	17.450
6	0.5244	17.972	17.940	17.810
7	0.5239	18.334	18.304	18.170
8	0.5241	18.881	18.845	18.710
9	0.5241	19.425	19.389	19.249
10	0.5240	19.968	19.934	19.789
11	0.5243	20.333	20.296	20.149
12	0.5240	20.515	20.476	20.329

**Table 7:** Robustness comparison on PEMS04 with 12-step input and output windows. MAE is averaged over all forecast steps; perturbed MAEs are averaged over ten paired corruption realizations, with percentage increases relative to each model’s clean-input MAE in parentheses. Gaussian noise is applied in the normalized input space.

Model	Clean MAE	Point Missing (50%)	Block Missing (6 Steps)	Gaussian Noise (σ=0.5)
DIST	17.890	20.359 (+13.8%)	20.878 (+16.7%)	19.071 (+6.6%)
ST-FGDIR	17.990	20.077 (+11.6%)	20.563 (+14.3%)	19.285 (+7.2%)

## Data Availability

The four public traffic forecasting benchmarks used in this study are METR-LA, PEMS-BAY, PEMS04, and PEMS08. The processed data splits, configuration files, experimental logs, source code, and implementation details are available from the corresponding author upon reasonable request.
